# The impact of computer–assisted technology on literacy acquisition during COVID-19-related school closures: Group–level effects and predictors of individual–level outcomes

**DOI:** 10.3389/fpsyg.2022.1001555

**Published:** 2022-12-02

**Authors:** Caroline G. Richter, Noam Siegelman, Kelly Mahaffy, Mark Van Den Bunt, Devin M. Kearns, Nicole Landi, John Sabatini, Kenneth Pugh, Fumiko Hoeft

**Affiliations:** ^1^Department of Psychology, University of Alabama at Birmingham, Birmingham, AL, United States; ^2^Department of Psychological Sciences, University of Connecticut, Mansfield, CT, United States; ^3^Haskins Laboratories, New Haven, CT, United States; ^4^Departments of Psychology and Cognitive and Brain Sciences, The Hebrew University of Jerusalem, Jerusalem, Israel; ^5^Department of Educational Psychology, Neag School of Education, Storrs, CT, United States; ^6^Department of Psychology and Institute for Intelligent Systems, University of Memphis, Memphis, TN, United States; ^7^Brain Imaging Research Center (BIRC), University of Connecticut, Storrs, CT, United States; ^8^Department of Psychiatry and Behavioral Sciences, and Weill Institute for Neurosciences, University of California, San Francisco, San Francisco, CA, United States; ^9^Department of Neuropsychiatry, Keio University School of Medicine, Tokyo, Japan

**Keywords:** COVID-19, early literacy skills, education technology, GraphoLearn, reading skills growth

## Abstract

**Introduction:**

The COVID-19 pandemic led to school closure and loss of in-person instruction during the 2019–2020 academic year across the United States, which had a profound impact on the reading development of beginning readers. In this study we tested if a research-informed educational technology (EdTech) program–GraphoLearn–could help alleviate the COVID-19 slide. We also sought to understand the profiles of children who benefitted most from this EdTech program.

**Methods:**

We tested participants’ (*N* = 172 K-2 children) early literacy skills using a standardized measure (STAR) before and after playing GraphoLearn, and used the pre to post difference as the dependent variable. We first compared children’s STAR actual and expected growth. Then we conducted a multiple regression analysis with data about engagement with GraphoLearn included as predictors. Additional predictors were extracted from GraphoLearn performance at study onset to assess children’s letter-sound knowledge, rime awareness, and word recognition.

**Results:**

The difference between actual average reading growth and expected growth in a regular school year was not statistically significant. This suggests that children in our sample seem to be gaining reading skills as expected in a regular school year. Our multiple linear regression model (which accounted for *R^2^* = 48% of reading growth) showed that older children, with higher baseline GraphoLearn word recognition, who played more units in a fixed number of days, made significantly more early literacy progress.

**Discussion:**

While lacking a control group, our preliminary results suggest that an EdTech program such as GraphoLearn may be a useful reading instructional tool during school shutdowns. In addition, our results suggest that practice with GraphoLearn was more effective and efficient when foundational instruction was already in place.

## Introduction

The ability to read is foundational for the engagement of individuals in contemporary society, allowing for success in school and the workplace, in addition to access to leisure activities (Castles et al., 2018; World Literacy Foundation, 2018). Given the critical importance of literacy skills, poor reading ability can lead to negative outcomes. Risk factors for poor reading ability include environmental factors such as growing up in poverty as well as biological factors such as genetic risk (Pennington, 2006; van Bergen et al., 2014; McGrath et al., 2020; Theodoridou et al., 2021; Erbeli et al., 2022). Limited reading abilities in adolescence and adulthood have been associated with a variety of poor outcomes including poorer educational attainment (OECD, 2016, 2019), psychosocial adjustment, job opportunities, and mental and physical health (National Reading Panel, 2000; Wilson et al., 2009). Given increased rates of high school dropout, anxiety, depression, and substance abuse, the cost associated with limited literacy abilities is high for both the individual and society (Wilson et al., 2009; Snowling and Melby-Lervåg, 2016; Hendren et al., 2018; World Literacy Foundation, 2018).

Providing preventative reading instruction that would reduce the probability of negative outcomes is therefore important. Research has shown that providing early reading instruction in kindergarten is more effective than general classroom practice, and waiting for 1 year to start early instruction can reduce its effectiveness by 25–50% (Ehri et al., 2001; Wanzek and Vaughn, 2007; Beddington et al., 2008; Wanzek et al., 2016). While early instruction is the goal, it is often difficult to carry out universally, particularly in diverse school settings with different resources. Considering the importance of evidence-based reading instruction for young learners (Castles et al., 2018), this study aimed to investigate the degree to which an at-home digital game-based reading program could help young children attain successful foundations for literacy.

Our study took place in a unique setting that is expected to result in particularly high levels of environmental risk: School closures due to the COVID-19 pandemic. During the early stages of the COVID-19 pandemic, access to high quality reading instruction was severely limited for many students, and using a game that could be played at home without the need for specialized instructors was particularly relevant. This context also allows for investigation of the efficacy of a reading instruction outside of the classroom, which if successful could allow for more access to high quality instruction materials even in communities who may have reduced resources in schools.

### COVID-19 pandemic and school closures

It is estimated that during the 2019–2020 academic year, at least 55.1 million students in 124,000 United States public and private schools experienced an unexpected closure of their K-12 school (Education Week, 2020). This closure had a profound impact on the development of beginning readers (grades K-2; Bao et al., 2020; Donnelly and Patrinos, 2021; Tomasik et al., 2021) particularly for those children in special education and/or at-risk for learning disorders who may find remote/online learning especially challenging (see Panagouli et al., 2021 for a systematic review). Indeed, extended periods without direct instruction, even a typical 3-month summer vacation period, can result in students losing the equivalent of 1 month of academic performance (Alexander et al., 2007a,b). The precise estimate of this well-known phenomenon, known as summer slide, varies depending on reading habits, socio-economic status (SES), grade-level, and level of performance including those in special education, language impairment and reading/learning disabilities (Cooper et al., 1996; Alexander et al., 2007a,b; Slates et al., 2012). Summer slide is especially important, as cumulative gaps based on experiences during school closure can account for up to 80% of the achievement gap between high and low achieving student (Alexander et al., 2007b). School closure due to the COVID-19 pandemic only widened this achievement gap (Domingue et al., 2021; Engzell et al., 2021; i-Ready, 2021; Amplify, 2022; PALS Report, 2022).

Young children who are in the early phases of reading seem to be more vulnerable to significant losses with reduced access to instruction (Bao et al., 2020; Wyse et al., 2020; Tomasik et al., 2021). Effective remediation to alleviate the COVID-slide is thus particularly important for young students who are learning foundational skills which must support more complex academic work for years to come. Practically, this leads to the prediction that those with weaker pre-reading or foundational skills will have longer lasting and more severe deficits. Bao et al. (2020) investigated kindergarten students over the pandemic and predicted that their rate of reading ability gain would decrease 66% compared to business-as-usal instruction. Wyse et al. (2020) also predicted greater learning loss for younger students. They reported that instruction is particularly important to support the reading progression in grades K-2, pointing to the lasting impact that early disruptions could have (Wyse et al., 2020). Similarly, a study conducted in Switzerland demonstrated that remote learning largely affected primary school students but not secondary school students (Tomasik et al., 2021). In particular, primary school students attending in-person school learned more than twice as fast as those learning remotely, but no differences were found for secondary students. Therefore, it is expected that the negative effects of school closure will be more evident for younger students. Recent reports confirm these predictions, with data consistently showing that the percentage of K-2 children who are being identified as at high risk for reading difficulties is higher than when compared to before the COVID-19 pandemic (Engzell et al., 2021; i-Ready, 2021; Amplify, 2022; PALS Report, 2022).

Exploring reading instructional programs for these younger students is particularly important, as it is clear that intervening early is the best way to promote success for struggling early readers. GraphoLearn, the game-based online program used in this study, addresses the need for widely accessible, research-based programs in the early years of reading instruction. GraphoLearn was designed to provide systematic, phonics-focused content using games with the hopes of creating an instructional tool that is both effective and fun for children (Richardson and Lyytinen, 2014). In this study, the game was provided to the families to be used at home with the goal of exploring literacy skills before and after playing GraphoLearn to see if it could help to mitigate the negative effects of school closures on reading abilities of K-2 grade students during COVID-19. This study is part of an effort to provide research-informed remote learning options to young children during school closures.

### GraphoLearn

There is good evidence that building high quality lexical representations for reading involves the complex integration of orthographic, phonological, and semantic features of words. In practice, this means that children must link the visual word form (the letters that spell a word such as ‘frog’), the sounds of the word (/f/ /r/ /au/ /g/), and the meaning of the word (a small, animal that says ribbit). Evidence shows that this integration depends on stable phonological, and orthographic-to-phonological decoding skills (Perfetti and Hart, 2002; Harm and Seidenberg, 2004). These skills are characteristically deficient (or lacking) in young struggling readers who have difficulty mapping letters to the phonetic constituents they represent, or trouble matching a letter form to the various sounds it might represent (Melby-Lervåg et al., 2012). Effective classroom instruction or remediation unsurprisingly depends on strengthening these component processing skills in a systematic fashion and there is a strong consensus on which processes need to be reinforced at progressive stages of learning (Wanzek and Vaughn, 2007; Galuschka et al., 2014; Wanzek et al., 2016).

Importantly, systematic phonics-based reading instruction is beneficial not just for struggling readers but also for typically developing readers (Brady, 2011). In recent years, there has been growing interest in how to teach these basic phonological and decoding skills using online reading instructional education technology (EdTech), but only few programs have been evaluated at scale. GraphoLearn is one: a well-studied program, delivered as an engaging computer game (Ahmed et al., 2020). GraphoLearn has been researched in German (e.g., Brem et al., 2010), Finnish (e.g., Saine et al., 2010), Portuguese (e.g., Carvalhais et al., 2020), and English (e.g., Kyle et al., 2013), among other languages (see McTigue et al., 2020 for a systematic review and meta-analysis). Generally, this research has shown inconclusive and mixed results about the game’s effectiveness, though vast differences in the age of participants, language abilities before starting the program, instruction timing, and other factors make these results difficult to interpret. Despite this, there is some evidence for the efficacy of the program and studies show that GraphoLearn is superior to various control conditions when the intervention group receives sufficient exposure to the game (Bhide et al., 2013; Kyle et al., 2013; Patel et al., 2018; Lassault et al., 2022).

Most of the previous studies evaluated the effectiveness of GraphoLearn while played in the school setting (86% per recent meta-analysis; McTigue et al., 2020). In the context of school closures during the pandemic, the possibility of having a game-like reading instruction that is engaging for children and can be played at home was particularly relevant. From a resources perspective, home administration is ideal, as it removes dependencies on school supports, resources and infrastructures, which can vary substantially. However, the home setting presents unique challenges, as the lack of structure or monitoring by a trained provider may decrease the level of engagement and compliance (Ronimus and Lyytinen, 2015; Landi and Cutting, 2017). While studies of GraphoLearn in laboratories, and controlled school settings, provide evidence of its efficacy, its performance when delivered at home has received less attention. For example, Ronimus and Lyytinen (2015) compared the effectiveness of GraphoLearn while played at home or in the school, showing that at school children played GraphoLearn more frequently, were more engaged and motivated, and teachers were more involved than parents. However, Mehringer et al. (2020) found that children at risk for dyslexia that played the Swiss-German version of GraphoLearn at home, showed improvements in their pseudoword reading skills. In this study we used the U.S. English version of GraphoLearn Rime, and children played the game at home under parental guidance.

### Individual-level differences in reading gains

While most studies have examined the impact of GraphoLearn at the group level (GraphoLearn group *vs* control group) to examine its efficacy, there is increasing interest in research that attempts to examine individual-level factors because of emerging evidence that GraphoLearn may not be as beneficial to all learners (McTigue et al., 2020). Understanding the characteristics of those who might benefit from the program is imperative. A recent meta-analysis emphasized the importance of adult interaction and support for the positive effect of the GraphoLearn to be achieved (McTigue et al., 2020), suggesting that GraphoLearn may be more effective for children in an environment that has more potential for such adult interactions. Although most studies of GraphoLearn suggest that short length of play time was a limitation and could explain why students learning was relatively limited, McTigue et al.’s (2020) meta-analysis indicated that duration was not a significant moderator of increases in reading ability.

Previous studies investigating the effectiveness of GraphoLearn have usually not taken an individual differences approach to understanding the characteristics of the students that respond better to the program. This approach is important, as it allows for more precision in understanding which characteristics might predispose a participant to positive response after playing the game. Therefore, in this study we also aimed to investigate participant-level characteristics to identify predictors of individual-level skills gains. An example of a study that took an individual-level approach was Hintikka et al. (2005). They investigated the predictiveness of pre-GraphoLearn skills for higher gains after playing GraphoLearn in Finnish. They found that the intervention was more effective for children with low phoneme awareness and more attention problems. However, since pre-intervention reading skills was close to zero, they did not investigate the effects of pre-literacy skills on intervention efficacy.

Previous research has demonstrated that knowledge of letter sounds (Muter et al., 2004) and rime awareness (Melby-Lervåg et al., 2012) are related to better response to intervention. Similarly, pre-intervention word reading skills are predictive of later reading outcomes (e.g., Vaughn et al., 2020). Therefore, in study we also investigated the predictive power of baseline knowledge of letter sounds, rime awareness, and word recognition skills to larger reading gains.

### Current study

The present study is a natural experiment conducted during the COVID-19 school closures in which we tested if GraphoLearn delivered at home, under parental guidance, is a potentially helpful tool to mitigate COVID-19 academic slide, and to identify which children benefit most from this EdTech program.

We explored two primary sets of questions. First: How much did children progress in their early literacy skills from baseline to after playing GraphoLearn (group-level), and how does this compare to the growth we would expect in a regular school year? Second: What are the significant predictors of reading growth (individual-level)? To address this question, we investigated several factors that we hypothesized would contribute to individual differences in reading gains from GraphoLearn, including progress made in the game, days played, early literacy skills at baseline, and SES.

## Materials and methods

### Participants

Participants included 172 children (93 females, 79 males) who were in Kindergarten (*n* = 80), Grade 1 (*n* = 75), or Grade 2 (*n* = 17) at study entry. At the end of the study, 55.6% of the children were in Grade 1 and 44.4% in Grade 2. The participants ranged in age from 4.30 to 8.77 years (*M* = 6.59 years, *M*dn = 6.56, *SD* = 0.86) at study entry (chronological age was not available for 12 participants). Data collection began in May 2020 and ended in November 2020.

Participation in the study required parents of the students to be able to understand enough English to consent and complete the questionnaires. In addition, participants had to understand the English instructions and have access to the necessary technology in their homes (i.e., a computer or a tablet connected to the internet). The participants’ racial/ethnic background was: 72.1% White, 14.5% multiracial, 4.1% Asian, 2.9% Black or African American, 1.2% American Indian or Alaskan Native, and 5.2% Other. Demographics information is summarized in Table 1. Regarding parental education, the highest level of education achieved for the primary caregiver was: high school degree or equivalent for 4.1%; some college (no degree) for 10.5%; Associate’s degree for 9.9%; Bachelor’s degree for 29.7%; professional degree for 2.9%; Master’s degree for 36.6%; and Doctorate degree (e.g., PhD, EdD) for 6.4%.

**Table 1:** Demographics and descriptive statistics.

Variable	*N*	%	Mean	Median	SD	Min–Max
Chronological age at study entry (years)	160		6.59	6.56	0.86	4.30–8.77
Grade at study entry						
Kindergarten	80	46.5				
Grade 1	75	43.6				
Grade 2	17	9.9				
Sex						
Female	93	54.1				
Male	79	45.9				
Race/ethnicity						
White	124	72.1				
Multiracial	25	14.5				
Asian/Asian American	7	4.1				
Black/African American	5	2.9				
American Indian or Alaskan native	2	1.2				
Other	9	5.2				
SES^a^	165		0	0.15	1	−2.49 to 2.61
Number of cars	165		1.84	2	0.4	0–2
Number of computers	165		2.24	3	0.87	0–3
Number of vacations in the last year	165		1.78	2	1.07	0–3
Median income by zip code ($)	165		83,632.34	76,159.00	32,662.01	28,965–201,528
Caregiver years of education	165		17.3	17	2.88	12–24
STAR early literacy score baseline	172		716.42	727.5	108.09	474–890
Kindergarten	80		681.81	689.5	109.3	474–877
Grade 1	75		737.05	749	98.68	478–890
Grade 2	17		788.24	819	85.85	588–887
STAR early literacy score post	172		731.18	739	102.37	395–890
Kindergarten	80		687.64	689	98.3	499–887
Grade 1	75		760.97	786	94.13	395–890
Grade 2	17		804.65	813	62.82	678–890
STAR early literacy score difference^b^	172		14.76	13.5	98.83	−425 to 272
Kindergarten	80		5.83	1.5	107.77	−289 to 272
Grade 1	75		23.92	17	94.32	−425 to 238
Grade 2	17		16.41	11	71.41	−140 to 172
GraphoLearn Number of units played	172		125.35	147.5	53.66	7–173
GraphoLearn days played	172		33.92	32.5	14.59	1–74
Number of days between baseline and post STAR assessment	172		47.28	43	17.64	7–164
Days from COVID-19 shutdown to pre-assessment^c^	172		114.7	104	43.09	61–255
GraphoLearn letter sounds^d^	170		0.8	0.83	0.13	0.17–1.00
GraphoLearn rime units^d^	171		0.4	0.35	0.26	0–1
GraphoLearn word recognition^d^	171		0.3	0.19	0.28	0–1

SES, socioeconomic status; ^a^SES is based on Principal Component Analysis of 5 questions; ^b^STAR Early Literacy Score difference = difference between STAR Early Literacy Score from baseline to post (after playing GraphoLearn); ^c^COVID-19 shutdown is defined as March 15, 2020; ^d^GraphoLearn performance at study onset are measured as proportion of correct responses.

Based on parental report, the majority of participants were developing typically (77.3%); 9.3% had speech-related problems; 5.3% had ADHD (one child with comorbid anxiety and two children with comorbid dyslexia); 1.7% had sensory processing issues; 2.3% had dyslexia; 1.2% had anxiety; 1.2% had autism spectrum disorders; 1.2% had developmental delay; and 0.6% (one child) was deaf with bilateral cochlear implants. Total number of participants that completed the baseline STAR Early Literacy assessment before starting GraphoLearn was 404. Only children that had at least some GraphoLearn playing time and both pre and post STAR Early Literacy assessment were included in the final analyzes.^1^ Mean STAR Early Literacy Scaled Score at baseline was not significantly different between included (*n* = 172; *M* = 716.42, SD = 108.09) and excluded participants (*n* = 232; *M* = 708.56, SD = 114.92), *t*(402) = −0.70, *p* = 0.486.

### Measures

#### STAR Early Literacy assessment

At baseline and after playing GraphoLearn, the STAR Early Literacy assessment (Renaissance Learning, 2022) was administered over Zoom and proctored by research staff. This is a nationally normed assessment that has been reviewed as reliable and valid. Generic reliability for grades *K*-3 range from 0.82–0.87, split-half reliability range from 0.83–0.90, and test–retest reliability range from 0.58–0.85 (Renaissance Learning, 2022).

This assessment consists of a 27-item adaptive assessment organized into three broad domains (Word Knowledge and Skills, Comprehension Strategies and Constructing Meaning, and Numbers and Operations) and 10 sub-domains (Alphabetic Principle, Concept of Word, Visual Discrimination, Phonemic Awareness, Phonics, Structural Analysis, Vocabulary, Sentence-Level Comprehension, Paragraph-Level Comprehension, and Early Numeracy). Children’s performance on each test item influenced the difficulty of the next item, meaning that the assessment was adaptive by item across broad and sub-domains. We provide definitions of the sub-domains as measured by STAR in the supplemental materials.

Based on these assessments, Early Literacy Scaled Score^2^ and Early Literacy Grade Equivalent scores are computed. Scaled scores are not standardized by age and grade and should be interpreted similarly to raw scores. A scaled score is calculated based on the difficulty of questions and the number of correct responses and is useful for comparing student performance over time and across grades. STAR Early Literacy scaled scores range from 300 to 900. A scaled score of 530 indicates sufficient kindergarten readiness (Renaissance Learning, 2022).

#### GraphoLearn

The first version of GraphoLearn, formerly known as GraphoGame (Lyytinen et al., 2009), was developed by reading researchers at the University of Jyvaskyla, for Finnish (a transparent language). Several versions were then developed for different languages with different levels of orthographic depth, including English (Richardson and Lyytinen, 2014). The GraphoGame terminology is also used for the commercial version of the game which is available through the App stores depending on the country and language. GraphoLearn, which was used in this study, is the research version of the game, which is always evolving, is not available through the App stores, and allow the researchers to collect extensive usage and user data for research. GraphoLearn fosters phonological awareness and teaches print-speech correspondences, thus mirroring best-practices for teaching reading in the classroom.

Between the two assessment timepoints, children independently played the U.S. English version of GraphoLearn Rime, version 12 (Richardson and Lyytinen, 2014), at home under parental guidance. Parents were instructed to encourage children to play GraphoLearn 20 min a day, 5 days per week, in two sessions of 10 min, with a break in-between the two sessions. Although all the parents received the same instruction, compliance (i.e., actual time spent playing GraphoLearn relative to prescribed) varied widely. For example, the number of days children played the game varied widely, ranging from 1 day to 74 days (*M* = 33.92, *SD* = 14.59).

GraphoLearn has a maximum of 173 units. Units are short modules of the game (the game ‘levels’) of multiple types such as choosing the word/letter/body that corresponds to a sound, combining sounds/letters into words, filling in missing sounds, etc. Among them are a maximum of seven assessments, dispersed uniformly between other units. Assessments are given to children at the end of a cluster of units to assess their learning of the material covered in those units. The complexity of the items in units is ordered such that at each level, the most frequent and regular mappings are introduced first (see GraphoLearn Scope and Sequence in supplemental materials). This allows for children to practice foundational skills before advancing to more difficult material. GraphoLearn is considered adaptive in that it requires children to achieve at least 80% accuracy on each assessment before introducing a new level of complexity. In practice, that should help to alleviate advancing to more complex content before foundational skills are in place.

We used children’s performance in the first assessment as additional measures of baseline literacy skills. These were extracted from GraphoLearn performance at study onset to assess children’s letter-sound knowledge, body-rime knowledge, and word recognition. Letter-sound knowledge included activities in which the child was asked to match the letter-sound pronounced by the game with the respective letter among different options. Rime units included activities in which the child was asked to match the sound pronounced by the game with one of the letter combinations options displayed in the screen (e.g., “ip,” “at,” “in,” “an”). Word recognition included activities in which the child was asked to match a word pronounced by the game (e.g., “can”) with the correct spelling among different options (e.g., “sin,” “pan,” and “can”). Split-half reliability for GraphoLearn first assessment for the participants included in our final sample was: 0.88 for letter-sound knowledge, 0.92 for rime units, and 0.97 for word recognition (all values are Spearman-Brown corrected).

In GraphoLearn Rime (Richardson and Lyytinen, 2014), players match auditory targets (i.e., sounds or words) to visual targets (e.g., single letters, letter sequences). The same stimuli and stimulus types are presented in multiple graphical settings to keep players engaged. For example, players select the ball containing the letter sequence corresponding to the auditorily presented rime among a series of balls falling from the top of the screen. The game provides trial wise feedback to guide players to select the correct mapping. GraphoLearn allows children to practice and reinforce lessons at their own pace and provides progress information that were used in our analyzes. Variables of interest used in following analyzes were: number of units played and number of days played.

#### Socioeconomic status

Socioeconomic status (SES) was assessed by five items: (1) number of cars in the household, (2) number of computers in the household, (3) number of vacations in the last 12 months, (4) median household income in zip code, and (5) years of education of primary caregiver. The first three questions were adapted from the Family Affluence Scale II (Boudreau and Poulin, 2009), which has been shown to be a valid measure of SES for a general population of 17,545 students in grades 7, 9, 10 and 12 in the Atlantic Provinces of Canada. Boudreau and Poulin (2009) reported that in 2001 the population of Atlantic Canada was relatively homogenous with greater than 95% of each of the provinces reporting Caucasian race. A score based on a Principal Component Analysis (PCA, first component) of these questions was created as our SES measure (see Supplementary Table S1, S2 of supplemental materials for PCA details).

#### Procedures

The study protocol was reviewed and approved by the university’s Institutional Review Board. Parents or legal guardians of all participants provided written informed consent. Participants for this study were generally recruited through fliers distributed by schools, *via* social media, or through the Haskins Global Literacy Hub website. In all cases, recruitment materials including general information about the project were made accessible to parents along with a link to an online form to express interest in the project. Once checking eligibility, researchers met with parents and children to explain the project, answer questions, and to complete the first STAR assessment. After completing the assessment, researchers provided download and sign-up instructions for GraphoLearn. For about 10% of the sample, researchers gave access codes to the teachers who in turn gave access codes to the students’ parents so they could receive the GraphoLearn, questionnaires, and various assessments. Caregivers were encouraged to monitor GraphoLearn administration through informational videos created by our research staff. Recommended strategies included having the child play the game in a quiet environment without other distractions, having an adult close to the child and available to help if they had any questions, and using a chart to track play and increase motivation. Parents were also instructed to use effort-based praise (i.e., “You did a great job working hard!”) as is consistent with studies conducted in person at the lab.

#### Statistical analyzes

To answer our first question about reading growth after playing GraphoLearn, we computed each participant’s literacy growth from pre- to post-GraphoLearn using STAR Early Literacy grade equivalent scores (i.e., measured in number of days). We then compared this growth in literacy skills (in days) to the number of (actual) days elapsed between pre- and post-assessments, using a paired sample t-test and a follow up Bayesian paired t-test. The rationale behind this analysis is that if participants made gains as expected in regular school years, we would observe no difference between their growth in reading skill and the number of days between assessments (and, in the corresponding Bayesian analysis, a Bayes Factor providing evidence in favor of the null hypothesis). If, however, they made lesser gains than expected in regular school years, there would be a significant (negative) difference between estimated reading growth in days and the number of actual days between sessions.

To answer our second question about the significant predictors of reading growth, multiple linear regression models were performed to identify the significant predictors of reading growth, while taking into account their baseline early literacy skills. The difference score, calculated by taking the difference of STAR pre and post testing, was used as the dependent variable in subsequent analyzes (STAR Early Literacy Score Difference = STAR post – STAR pre). Then, we predicted the variability in these difference scores, while controlling for variability in the baseline early literacy scores, therefore we are predicting residualized gains.^3^ Independent variables were: number of units played in the GraphoLearn game, number of days played, and GraphoLearn’s measures of literacy performance at baseline. In addition to baseline STAR Early Literacy Score, number of days between baseline and post STAR assessment, days from COVID-19 shutdown to pre-assessment (defined as March 15, 2020), and SES were entered as covariates. All assumptions of multiple linear regression analyzes were met (i.e., linearity, homoscedasticity, independence, and normality). Cohen’s *f^2^* was used to measure effect size (0.02 = small effect, 0.15 = medium, 0.35 = large; Cohen, 1988).

## Results

### Average early literacy growth

Descriptive statistics for variables included in the analyzes are presented in Table 1. Between the two assessment points, children made an average growth of 0.128 years (*SD* = 0.84, range: −2.30 – 2.70) on STAR Early Literacy grade equivalent scores. This corresponds to an average growth of 46.9 days (0.128*365 = 46.9 days) in a regular school year (*M* = 46.90; *SD* = 306.54, range: −839.50 – 985.50). The time that actually elapsed between the two timepoints was 47.28 days on average (*SD* = 17.64, range: 7–164). A paired sample t-test revealed that the difference between the actual time elapsed and the observed growth was not statistically significant, *t*(171) = 0.02, *p* = 0.987. To confirm that the lack of difference between the observed growth and time elapsed reflects a true null result, we also ran a Bayesian paired t-test (using the ttestBF() function from the BayesFactor R package, Morey et al., 2018, and using default priors). This analysis revealed strong support for the null hypothesis, BF10 = 0.085, meaning that the data are 1/0.085 = 11.7 times more likely under the null hypothesis (i.e., no difference between time elapsed and growth) than under the alternative hypothesis (i.e., some difference between time elapsed and growth).

In that sense, children in our sample seem to be gaining reading skills as expected in a regular school year (on average), suggesting that GraphoLearn might have had a positive mitigating effect on the detrimental impact of COVID-19-related school closures. However, there was considerable variability with some children scoring lower after playing GraphoLearn and others scoring much higher (see Table 1). Given this pattern, understanding which variables contributed to reading growth at the individual level is imperative. This question was addressed using multiple regression in the central analyzes below.

### Predictors of early literacy growth

Bivariate correlations among the measures included in the regression analyzes are presented in Table 2. The pattern of correlations with chronological age and grade was very similar. Since chronological age was missing for 12 participants, grade was controlled for in the multiple regression analyzes. Correlations among sex and the other measures included in the study were not significant, therefore sex was not included in the multiple regression analyzes.

**Table 2:** Bivariate correlations among the measures included in the study.

Variable	*N*	2	3	4	5	6	7	8	9	10	11	12	13
1. Chronological age at study entry	160	0.686**	−0.05	0.475**	−0.056	0.254*	−0.296**	−0.075	−0.126	0.335**	0.266*	0.284**	−0.133
2. Grade at study entry	172		−0.072	0.328**	0.067	0.132	−0.344**	−0.189	0.145	0.275**	0.316**	0.337**	0.031
3. Sex^a^	172			−0.026	−0.009	0.075	0.091	0.011	0.074	0.033	−0.018	−0.019	0.088
4. STAR early literacy score pre	172				−0.513**	0.354**	−0.332**	−0.114	−0.031	0.437**	0.401**	0.510**	−0.13
5. STAR early literacy score difference	172					−0.121	−0.077	0.09	0.083	−0.079	−0.024	−0.019	0.127
6. GraphoLearn number of units played	172						0.303**	−0.089	0.011	0.326**	0.312**	0.243*	−0.025
7. GraphoLearn days played	172							0.093	−0.005	−0.253*	−0.257*	−0.386**	0.113
8. Number of days between baseline and post STAR assessment	172								−0.099	−0.166	−0.179	−0.166	0.001
9. Days from COVID-19 shutdown to pre-assessment	172									0.003	0.044	0.039	0.323**
10. GraphoLearn letter sounds	170										0.479**	0.291**	−0.122
11. GraphoLearn rime units	171											0.434**	−0.057
12. GraphoLearn word recognition	171												−0.037
13. SES	165												

*p < 0.01; **p < 0.001. ^a^Females were coded as 0 and males as 1.

There was a strong negative correlation between the dependent variable (i.e., STAR Early Literacy Score pre to post change) and its autoregressor (i.e., STAR Early Literacy Score at baseline; *r* = −0.51, *p* < 0.001).^4^ STAR Early Literacy Score at baseline was positively and strongly correlated with GraphoLearn Word Recognition at study entry (*r* = 0.51, *p* < 0.001), GraphoLearn Letter Sounds (*r* = 0.44, *p* < 0.001), and GraphoLearn Rime units (*r* = 0.40, *p* < 0.001), suggesting that STAR and the GraphoLearn assessments reflect some degree of shared variance in reading skills (although they most likely also tap into different sub-components; see more on this point in the General Discussion). STAR Early Literacy Score at baseline was also positively and moderately correlated with number of units played in GraphoLearn (*r* = 0.35, *p* < 0.001), indicating that children with better reading skills at baseline played through more units of GraphoLearn. In contrast, STAR Early Literacy Score at baseline was negatively and moderately correlated with number of days playing GraphoLearn (*r* = −0.33, *p* < 0.001), indicating that children with lower reading skills at baseline took more days to play the game. The three variables collected from GraphoLearn at baseline (letter-sound knowledge, rime awareness, and word recognition) were positively and moderately correlated with each other (*r’*s between 0.29 and 0.48).

To identify significant predictors of reading growth (i.e., STAR Early Literacy Score pre to post change), we began by fitting a multiple regression model (Model 1) that included the following independent variables: STAR Early Literacy Score at baseline (autoregressor), number of units played in GraphoLearn, number of days played, number of days between baseline and post STAR assessment, and days from COVID-19 shutdown (defined as March 15, 2020) to pre-assessment (Model 1). As noted above, since STAR Early Literacy Scores at baseline was controlled for in all models, we were predicting residualized change scores. Model 1 explained 45% of the variance in STAR Early Literacy Score difference, *R^2^* = 0.45, adjusted *R^2^* = 0.43, *F*(6, 155) = 21.52, *p* < 0.001 (see Table 3). Older kids had greater residualized gains on average when controlling for other factors (*B* = 27.12, *p* = 0.006, Cohen’s *f^2^* = 0.05). Lower STAR Early Literacy Score at baseline (*B* = −0.70, *p* < 0.001, Cohen’s *f^2^* = 0.77), greater number of units (*B* = 0.50, *p* < 0.001, Cohen’s *f^2^* = 0.10) and a smaller number of days played in GraphoLearn (*B* = −2.34, *p* < 0.001, Cohen’s *f^2^* = 0.15) were predictive of positive residualized STAR change scores. It is important to note that when including both number of units and number of days played in GraphoLearn in the same model we were investigating the number of units per fixed playing time; and playing time per fixed number of units. These results indicate that children who played more units in a fixed number of days made significantly more reading progress than those who played less units in the same number of days. In contrast, children who took more days to play the same number of units made significantly less reading progress.

**Table 3:** Multiple regression analyzes predicting STAR Early Literacy score difference.

Predictor	*B*	*t*	value of *p*	95% CI for *B*	semi-partial *r*	Cohen’s *f^2^*
**Model 1**						
Constant	482.63	8.9	<0.001	[375.55, 589.70]		
Grade at study entry	27.12	2.76	0.006	[7.75, 46.50]	0.16	0.05
STAR early Literacy score baseline	−0.7	−10.92	<0.001	[−0.83, −0.58]	−0.65	0.77
GraphoLearn: units played	0.5	3.88	<0.001	[0.25, 0.75]	0.23	0.1
GraphoLearn: days played	−2.34	−4.91	<0.001	[−3.28 to −1.40]	−0.29	0.15
Days between pre and post STAR	0.65	2.03	0.044	[0.02, 1.29]	0.12	0.03
Days from COVID-19 shutdown to pre STAR	0.06	0.48	0.634	[−0.20, 0.33]	0.03	<0.01
*R^2^* = 0.45, adjusted *R^2^* = 0.43, *F*(6, 155) = 21.52, *p* < 0.001						
**Model 2 (Final model)**						
Constant	461.24	7.64	<0.001	[341.90, 580.58]		
Grade at study entry	22.45	2.34	0.021	[3.46, 41.45]	0.13	0.04
STAR early literacy score baseline	−0.78	−11.75	<0.001	[−0.92, −0.65]	−0.68	0.91
GraphoLearn: units played	0.34	2.5	0.014	[0.07, 0.61]	0.14	0.04
GraphoLearn: days played	−1.69	−3.39	0.001	[−2.67, −0.71]	−0.2	0.08
Days between pre and post STAR	0.78	2.49	0.014	[0.16, 1.41]	0.14	0.04
Days from COVID-19 shutdown to pre STAR	0.06	0.48	0.63	[−0.20, 0.32]	0.03	<0.01
GraphoLearn: letter Sounds	54.32	1.11	0.27	[−42.62, 151.26]	0.06	0
GraphoLearn: rime Units	24.01	0.91	0.366	[−28.28, 76.30]	0.05	0.01
GraphoLearn: word recognition	72.42	2.88	0.004	[22.81, 122.04]	0.17	0.06
*R^2^* = 0.50, adjusted *R^2^* = 0.47, *R^2^* change = 0.04, *F* change (3, 152) = 4.17, *p* = 0.007						
**Model 3**						
Constant	462.91	7.67	<0.001	[343.66, 582.16]		
Grade at study entry	21.62	2.25	0.026	[2.60, 40.65]	0.13	0.03
STAR early literacy score baseline	−0.78	−11.66	<0.001	[−0.91, −0.65]	−0.67	0.9
GraphoLearn: units played	0.34	2.53	0.012	[0.08, 0.61]	0.15	0.04
GraphoLearn: days played	−1.74	−3.49	0.001	[−2.73, −0.75]	−0.2	0.08
Days between pre and post STAR	0.78	2.47	0.015	[0.15, 1.40]	0.14	0.04
Days from COVID-19 shutdown to pre STAR	0.01	0.07	0.941	[−0.26, 0.28]	< 0.01	<0.01
GraphoLearn letter sounds	58.58	1.19	0.235	[−38.53, 155.69]	0.07	<0.01
GraphoLearn rime units	23.54	0.89	0.375	[−28.71, 75.78]	0.05	<0.01
GraphoLearn word recognition	71.42	2.85	0.005	[21.83, 121.02]	0.16	0.05
SES	6.72	1.15	0.251	[−4.79, 18.23]	0.07	0.1
*R^2^* = 0.50, adjusted *R^2^* = 0.47, *R^2^* change = 0.004, *F* change(1, 151) = 1.33, *p* = 0.251						

*N* = 162; CI, confidence interval.

**Figure 1:**
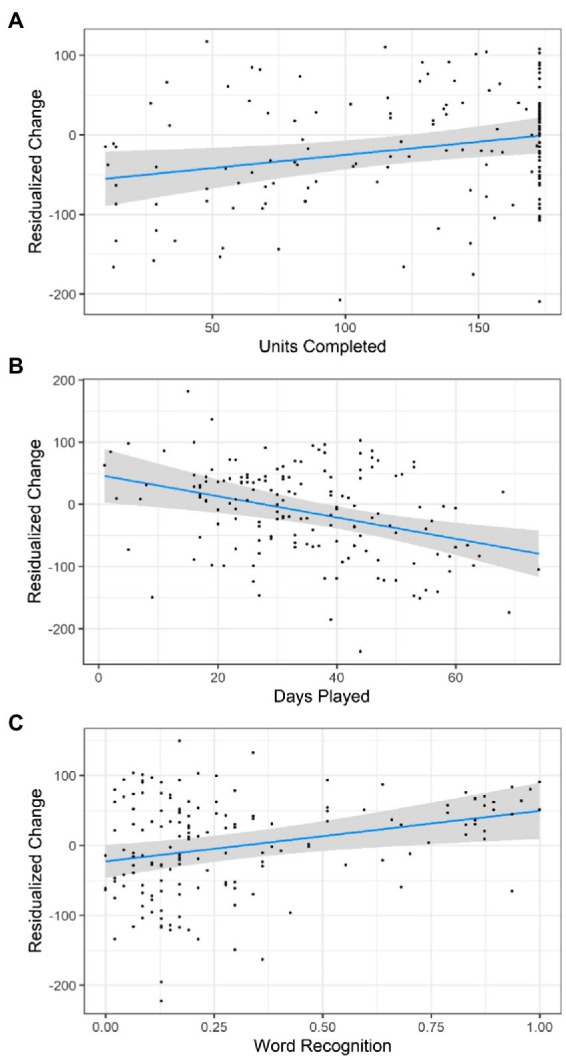
Significant predictors of STAR early literacy score difference. The effect of number of units completed **(A)**, number of days played **(B)**, and word recognition skill at the beginning of the GraphoLearn program **(C)** on residualized STAR Early Literacy change scores. The plots present conditional effects, controlling for all predictors included in the model in Table 3. Older kids had greater residualized gains on average when controlling for other factors. Plots created using the visreg R package (Breheny and Burchett, 2017).

In Model 2, scores in GraphoLearn Letter Sounds, GraphoLearn Rime units, and GraphoLearn Word Recognition were added to Model 1 (see Table 3). Model 2 accounted for significantly more variance in STAR Early Literacy Score difference than did Model 1, *R^2^* = 0.50, adjusted *R^2^* = 0.47, *R^2^* change = 0.04, *F* change(3, 152) = 4.17, *p* = 0.007. Besides the significant contribution of the three previous independent variables (i.e., STAR Early Literacy Score at baseline, number of units and number of days played in GraphoLearn, all remaining significant in Model 2), number of days between baseline and post STAR assessment (*B* = 0.78, *p* = 0.014, Cohen’s *f^2^* = 0.04) and word recognition skills at baseline (*B* = 72.41, *p* = 0.004, Cohen’s *f^2^* = 0.06) made significant unique contributions to the model. These results indicate that children who had better word recognition skills at study entry were able to gain significantly more reading skills after playing GraphoLearn.^5^ Figure 1 shows plots of the significant predictors of Model 2. The plots present conditional effects, controlling for all predictors included in the model in Table 3. Another visualization of the same effects are provided in Supplementary Figure S1, which presents the conditional effects shown in Figure 1 using a two-dimensional space where predicted STAR Early Literacy change scores are depicted as a function of number of units completed and number of days played.

Finally, we entered SES as the last independent measure in Model 3 (see Table 3). The addition of SES did not result in a significant increase in the amount of variance explained in STAR Early Literacy Score difference, *R^2^* = 0.50, adjusted *R^2^* = 0.47, *R^2^* change = 0.004, *F* change(1, 151) = 1.33, *p* = 0.251.

## Discussion

The present study examined whether GraphoLearn, delivered at home, under parental guidance, could be a potentially helpful tool to mitigate the COVID-19 slide in reading skills. In addition, we also investigated who are the children who benefit the most from this EdTech program. We found that on average, children’s literacy skills grew as expected when playing GraphoLearn. Regarding our second question, three major variables emerged as significant predictors of reading growth: number of GraphoLearn units played, number of days played, and word recognition skills at baseline. Thus, our preliminary results suggest that GraphoLearn was, on average, a helpful tool for mitigating the detrimental impact of COVID-19-related school closures on early literacy skill acquisition, but this pattern varied according to individual-level characteristics. We discuss these two central findings below.

### Average early literacy growth

While this study was taking place, children were not in traditional classroom settings due to COVID-19 school closures. However, in many situations teachers worked with children online and sent materials to children’s homes to be completed with parents’ support. Unfortunately, information about participants schools’ protocols during the pandemic was not available. Despite school’s instruction efforts, for this age group (K-2) it would be expected that the rate of reading ability gain would decrease when compared to a business-as-usal scenario (Bao et al., 2020; Wyse et al., 2020; Tomasik et al., 2021). As highlighted in the introduction, both prediction studies (Bao et al., 2020; Wyse et al., 2020; Tomasik et al., 2021) and recent reports (i-Ready, 2021; Amplify, 2022; PALS Report, 2022) confirm that children’s growth in word reading skills decreased, on average, due to COVID-19 closures. For example, Amplify (2022) using the Dynamic Indicators of Basic Early Literacy Skills (DIBELS) reported that in the year 2020–21 an average of 41% K-2 students was far behind (in need of intensive intervention), in comparison with 27% before the COVID-19 pandemic (2019–20). Similarly, for students in Grades 2, an average of 33% were reported to be below grade level in reading in the Fall 2021 compared to 24% based on historical averages (i-Ready, 2021). Finally, PALS Report (2022) reported that the percentage of K-2 students below the benchmark rates in a Phonological Awareness Literacy Screening increased from 21.3% in 2019 to 34.9% in 2021 (after the COVID-19 pandemic).

In our analyzes, we compared participants’ reading growth to the expected growth in STAR Early Literacy grade equivalent scores in regular (non-COVID) years. The result of our analysis provided evidence for a lack of a difference between observed and expected growth. These findings suggest that, on average, children benefited from playing GraphoLearn at home, which is consistent with previous studies showing that GraphoLearn is an effective program (Bhide et al., 2013; Kyle et al., 2013; Patel et al., 2018).

Despite these positive findings, our comparison has limitations. First, we do not have information about expected reading growth in STAR Early Literacy during COVID-19 school closures. Second, this comparison assumes a linear growth throughout the year, which previous studies have demonstrated is not true. In particular, in certain months of the year, depending on grade level, there is more or less growth in reading and related subskills and this information is not available from STAR assessments (Renaissance Learning, 2022). Finally, recent reports (Domingue et al., 2021; Engzell et al., 2021; Amplify, 2022; PALS Report, 2022) suggest that losses are larger among students from less-educated homes. So these preliminary findings need to be interpreted cautiously, taking into consideration that the majority of our sample was White (72%) and from highly educated families (76% of primary caregivers had at least a Bachelor’s degree).

### Predictors of early literacy growth

Our preliminary results also provide evidence that children with better word recognition skills at baseline, as measured by GraphoLearn, showed greater change in overall early literacy skills, benefitting more from playing GraphoLearn than those with lower word recognition abilities at baseline, after taking the other variables included in the model into account. In particular, children with higher word recognition scores at baseline showed greater improvement in STAR (early literacy skills) while taking into account their starting point in the same measure (i.e., word recognition positively predicted residualized change in literacy skills). Along these lines, we observed that practice with GraphoLearn was more effective and efficient when foundational instruction was already in place. That is, children with higher word recognition skills at baseline had enough previous instruction to use GraphoLearn to practice, maintain, and extend their skills, while others did not, hence struggled more (took longer to complete units), leading to less efficient learning. These findings are information for future research that aims at modifying and extending the GraphoLearn to account for those students who are lacking those initial word recognition skills.

These results are consistent with the Matthew effects in reading, that children who started playing the game with better word recognition skills at baseline are able to learn more from the program and their early literacy growth is therefore higher than those children who started with lower word recognition skills (Stanovich, 1986). In other words, the “rich-get-richer” (Stanovich, 1986). But beyond the typical Matthew effect, our results suggest a *specific* positive relation between word recognition skills at baseline and early literacy skills growth as a result of the EdTech program, observed while controlling for other variables including overall Early Literacy skills at baseline. Evidence for the specificity of this link comes from the fact that other GraphoLearn assessment components (i.e., letter-sound knowledge and body-rime knowledge) were not significant predictors of early literacy growth after controlling for pre-early literacy skills. The specific link between baseline word recognition skill and early literacy growth suggests that GraphoLearn is particularly effective for early readers who already have some word recognition abilities, and potentially less so for those who have yet to develop these (relatively advanced) skills.

In summary, our preliminary results suggest that GraphoLearn is effective because it teaches young children to map letters to the phonetic constituents they represent in a systematic way, which is key to learning to read in alphabetic languages (Melby-Lervåg et al., 2012; Castles et al., 2018). GraphoLearn appears to be an effective online reading instructional education technology that teaches these basic phonological and decoding skills, in the context of school-closures. However, with this study we were unable to identify the exact GraphoLearn mechanism that led to better early literacy skills. Future studies could manipulate various potential GraphoLearn processes responsible for a positive reading effect to better identify the specific processes that underly this effect. These preliminary results can be expanded to suggest that EdTech games that are research and evidence-based and follow EdTech best practices, can have a positive impact in children’s development. In particular, our results add to the extant GraphoLearn literature that has mostly emphasized the use of the game in school settings (McTigue et al., 2020) to suggest that playing the game at home under parental guidance, might lead to significant early literacy skills improvements (e.g., Ronimus and Lyytinen, 2015; Mehringer et al., 2020).

### Limitations and future directions

The results of the present study should be interpreted in the context of certain limitations. First, while some previous studies demonstrate the efficacy of the GraphoLearn program compared to a control condition (Bhide et al., 2013; Kyle et al., 2013; Patel et al., 2018), all the participants included in this study were invited to play the GraphoLearn at home and were given access to the license for free, meaning that there was no control group in our design. Considering the unprecedent learning losses due to COVID-19 related school closures, it would not have been appropriate to deny access to this resource to interested families. Therefore, given these ethical considerations, we were unable to have a group comparison during the COVID-19 pandemic that would allow us to better evaluate the program’s efficacy relative to a group of children who were not instructed to play the game (using either an active or passive control group).

Second, the absence of a reliable measure to index caregiver involvement with their children while they were playing GraphoLearn is another important limitation. This is even more relevant when we consider recent findings by McTigue et al. (2020) which suggest the importance of adult interaction and support for positive effects of GraphoLearn to be realized. Although families were encouraged to supervise children while they were playing the game, information on if they did or did not, what type of praise they used, and how often they praised children was not available. While this factor would add another interesting dimension to our models, our work provides evidence that children who do play the game can gain early literacy skills.

Third, according to Green (1991) in order to detect a small effect size in a multiple regression model with 10 predictors, a sample size of at least 788 participants would be required.^6^ Although we cannot determine with precision if the effect size of SES would be considered small in this unique setting, previous studies have found, in general, a small effect of SES on response to reading interventions (D’Angiulli et al., 2004; Noble et al., 2006; Romeo et al., 2018; Dolean et al., 2019). Future studies should address these limitations by focusing on evaluating the effectiveness of GraphoLearn through a randomized controlled trial, while accounting for the problems observed in this study. These include monitoring caregiver involvement and inclusion of a larger number of participants that would allow for detection of smaller effect sizes such as the one that could have been played by SES. In addition, although we made efforts to include a diverse sample of participants (i.e., we contacted schools and teachers so they could make GraphoLearn accessible to their students – but for the vast majority of the sample, parents contacted us demonstrating interest in participating in our study) we did not have a lot of variability in our SES measure and, as previously noted, most of our participants were White and well-educated. Importantly, considering the demographics of our participants, they may have access to other resources in their house beyond GraphoLearn which might also be supporting their early literacy skills (Burgess et al., 2002; Aikens and Barbarin, 2008; O’Donnell, 2008; Froiland et al., 2013).

Despite these limitations, the current study provides preliminary evidence that playing GraphoLearn can help maintain typical literacy growth, particularly during a disruption of typical school activities. While large decreases in early literacy skills were expected for young children due to COVID-19 related school shutdowns, on average, children in our sample increased early literacy knowledge over this period. However, there is no claim that the program will be substitute for individualized, and expert-supervised, reading remediation. Moreover, individual differences that predict positive changes in early literacy skill, such as the number of units and days played in GraphoLearn and baseline word recognition knowledge provide useful metrics to increase understanding of which students may thrive using a program such as GraphoLearn or a similar EdTech game.

## Data availability statement

The raw data supporting the conclusions of this article will be made available by the authors, without undue reservation.

## Ethics statement

The studies involving human participants were reviewed and approved by University of Connecticut. Written informed consent to participate in this study was provided by the participants’ legal guardian/next of kin.

## Author contributions

CR wrote the first draft of the manuscript. NS and KM organized the database and wrote sections of the manuscript. KM supervised data collection. NS and CR performed the statistical analysis. FH, KP, JS, NL, DK, and MB contributed to conception and design of the study. All authors contributed to manuscript revision, read, and approved the submitted version.

## Funding

This project was supported by the National Science Foundation 20-052 COVID-19, RAPID mechanism; #2029373 and the Tremaine Foundation. FH was also funded by the National Science Foundation #2152202, NICHD R01HD094834, NICHD R01HD096261, and NCCIH U24AT011281. NL and KM were also funded by NIH R01HD101842. The content is solely the responsibility of the authors and does not necessarily represent the official views of the National Institutes of Health.

## Conflict of interest

The authors declare that the research was conducted in the absence of any commercial or financial relationships that could be construed as a potential conflict of interest.

## Publisher’s note

All claims expressed in this article are solely those of the authors and do not necessarily represent those of their affiliated organizations, or those of the publisher, the editors and the reviewers. Any product that may be evaluated in this article, or claim that may be made by its manufacturer, is not guaranteed or endorsed by the publisher.

## Footnotes

^1^We conducted the same set of analyses including only the participants that were developing typically based on parent report and the pattern of results was maintained. Children were considered to be developing typically if their parents answered "none" for the question "Does your child have any identified or suspected developmental, medical, or psychological conditions?"

^2^We are using the term "Scaled Score" for consistency with STAR terminology. STAR uses IRT-scaling across items, not standardization over chronological age or grade.

^3^Another approach would be to predict STAR post while controlling for STAR pre. When these analyses were performed, all significant predictors remained significant and all non-significant predictors remained non-significant. See Table S3 in supplemental materials.

^4^The correlation between STAR pre and STAR post was strong and positive (*r* = 0.56, *p* < 0.001). But as expected (see Clinical Trial Design and Analysis (2022) for a tutorial), the STAR change was correlated with the STAR baseline just as much as STAR post score, just in the opposite direction.

^5^To confirm that the significant effects are not driven by a small number of extreme observations, we conducted analyses removing the top 10% and the bottom 10% performers on STAR pre and results remained the same (i.e., all significant predictors remained significant and all non-significant predictors remained non-significant).

^6^Our study is sufficiently powered to detect medium and large effect sizes.
