# Supplementary material for: The impact of computer–assisted technology on literacy acquisition during COVID-19-related school closures: Group–level effects and predictors of individual–level outcomes
Source: Front Psychol. 2022 Dec 2;13:1001555. doi: 10.3389/fpsyg.2022.1001555 (PMC9755674; doi:10.3389/fpsyg.2022.1001555)
Supplement: Supplementary file 1 [file Data_Sheet_1.zip › Table 1.DOCX]

Supplementary Material

**1 Definitions of the sub-domains as measured by STAR Early Literacy Assessment**

Alphabetic Principle assesses a student’s knowledge of letter names, alphabetic letter sequences, and the sounds associated with letters. Concept of Word assesses a student’s understanding of print concepts regarding written word length and word borders and the difference between words and letters. Visual Discrimination assesses a student’s ability to differentiate both upper and lowercase letters, identify words that are different, and match words that are the same. Phonemic Awareness assesses a student’s understanding of rhyming words; blending and segmenting word parts and phonemes; isolating and manipulating initial, final, and medial phonemes; and identifying the sounds in consonant blends. Phonics assesses a student’s understanding of short, long, and variant vowels and other vowel sounds; initial and final consonants; consonant blends and digraphs; consonant and vowel substitution; and identification of rhyming words and sounds in word families. Structural Analysis assesses a student’s understanding of affixes and syllable patterns in decoding and identification of compound words. Vocabulary assesses a student’s knowledge of high-frequency words, regular and irregular sight words, multi-meaning words, words used to describe categorical relationships, position words, synonyms and antonyms. Sentence-Level Comprehension assesses a student’s ability to identify the meaning of words in contextual sentences. Paragraph-Level Comprehension assesses a student’s ability to identify the main topic of text and the ability to answer literal and inferential questions after listening to or reading text. Early Numeracy assesses a child’s ability to identify and name numbers; understand number-object correspondence; complete sequences; compose and decompose groups of up to ten; and compare sizes, weights, and volumes (Renaissance Learning, 2022). Overall, then, the STAR Early Literacy test assesses pre-reading subskills that are important for the development of future literacy skills.

# 2 Supplementary Figures and Tables

**Table S1**

*Factor Loadings and Communalities for Principal Component Analysis (first component) to Create the Socioeconomic Status Composite* (*n* = 165)

|  | Factor loading | |  |
| --- | --- | --- | --- |
|  | 1 | 2 | Communality |
| Number of cars | 0.72 |  | 0.64 |
| Number of computers | 0.60 |  | 0.57 |
| Number of vacations in the last year | 0.49 | 0.32 | 0.36 |
| Median income by zip code ($) |  | 0.77 | 0.34 |
| Caregiver years of education | 0.33 | 0.68 | 0.58 |

*Note.* Varimax Rotation with Kaiser Normalization.

**Table S2**

*Eigenvalues, Percentages of Variance and Cumulative Percentages for Factors - Socioeconomic Status Composite* (*n* = 165)

| Factor | Eigenvalue | % of Variance | Cumulative % |
| --- | --- | --- | --- |
| 1 | 1.32 | 26.41 | 26.41 |
| 2 | 1.18 | 23.55 | 49.96 |
| 3 | 0.96 | 19.18 | 69.14 |
| 4 | 0.78 | 15.67 | 84.81 |
| 5 | 0.76 | 15.19 | 100.00 |

**Figure S1.** *Predicted Change Scores in STAR Early Literacy (Color Scale): Conditional Effects of Number of Units Completed (x-axis) and Number of Days Played (y-axis). Plot Created Using the visreg R Package (Breheny & Burchett, 2017).*


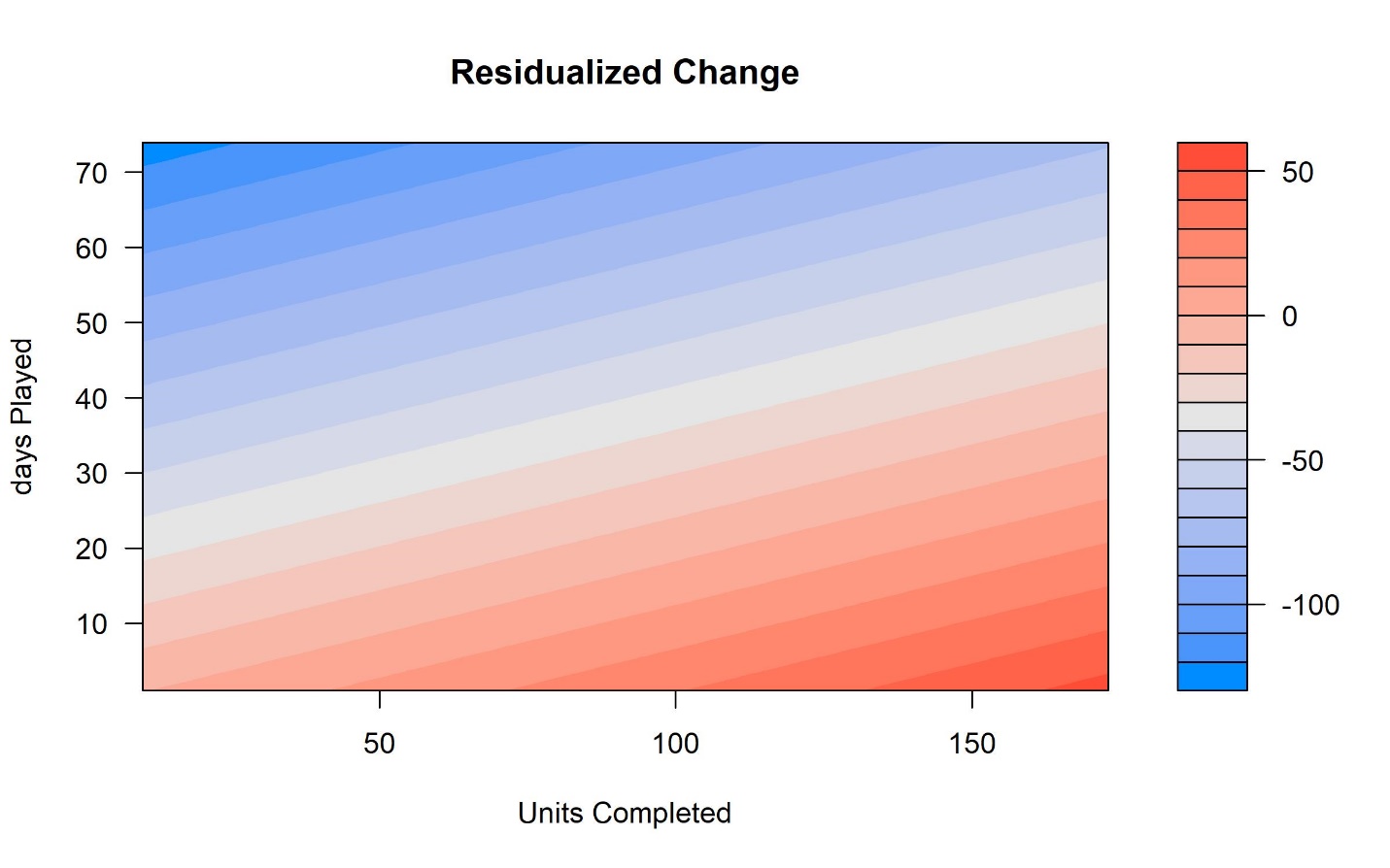


**Table S3**

*Multiple Regression Analyses Predicting STAR Early Literacy Score Post*

| Predictor | *B* | *t* | *p*-value | 95% CI for *B* | semi-partial *r* | Cohen’s *f^2^* |
| --- | --- | --- | --- | --- | --- | --- |
| **Model 3** |  |  |  |  |  |  |
| Constant | 462.91 | 7.67 | < .001 | [343.66, 582.16] |  |  |
| Grade at study entry | 21.62 | 2.25 | .026 | [2.60, 40.65] | .12 | 0.03 |
| STAR Early Literacy Score Baseline | 0.22 | 3.30 | .001 | [0.09, 0.35] | .18 | 0.07 |
| GraphoLearn: Units played | 0.34 | 2.53 | .012 | [0.08, 0.61] | .14 | 0.04 |
| GraphoLearn: Days played | -1.74 | -3.49 | .001 | [-2.73, -0.75] | -.19 | 0.08 |
| Days between pre and post STAR | 0.78 | 2.47 | .015 | [0.15, 1.40] | .14 | 0.04 |
| Days from COVID-19 shutdown to pre STAR | 0.01 | 0.07 | .941 | [-0.26, 0.28] | < .01 | < 0.01 |
| GraphoLearn Letter Sounds | 58.58 | 1.19 | .235 | [-38.53, 155.69] | .07 | 0.01 |
| GraphoLearn Rime units | 23.54 | 0.89 | .375 | [-28.71, 75.78] | .05 | 0.01 |
| GraphoLearn Word Recognition | 71.42 | 2.85 | .005 | [21.83, 121.02] | .16 | 0.05 |
| SES | 6.72 | 1.15 | .251 | [-4.79, 18.23] | .06 | 0.13 |
| *R^2^* = .54, adjusted *R^2^* = .51, *F*(10, 151) = 17.69, *p* < .001 | | | | | | |

*Note. N* = 162. CI = confidence interval.
