# Supplementary material for: The impact of computer–assisted technology on literacy acquisition during COVID-19-related school closures: Group–level effects and predictors of individual–level outcomes
Source: Front Psychol. 2022 Dec 2;13:1001555. doi: 10.3389/fpsyg.2022.1001555 (PMC9755674; doi:10.3389/fpsyg.2022.1001555)
Supplement: Supplementary file 1 [file Data_Sheet_1.zip › Table 2.DOCX]

## **How Grapholearn works:** By playing the game, children learn first the basic letters and their sounds. Through a series of levels, they gradually move on to short and increasingly longer words. GraphoLearn dynamically adapts the difficulty level to the child’s unique ability level.

**Goals:** Students must reach 80% proficiency on each level to progress forward. Students will earn rewards as they move through each level.

| **Scope and Sequence** |
| --- |

| **Level** | **Focus** |
| --- | --- |
| **Stream 1-5** | **Focus**: All letter sounds are introduced. Short vowels and word families are introduced.  ***Examples****: It, Pad, Mop, Jet, Cat* |
| **Stream 6** | **Focus**: Consonant digraphs: sh and ch  ***Examples****: dash, splash, chop, cash* |
| **Stream 7** | **Focus**: Glued (welded) sounds: ing, ang, ong  ***Examples****: Along, Thing, Rang* |
| **Stream 8** | **Focus**: Vowel teams, glued (welded) sounds, and word families: -ill, -ank, ea, ll, nk  ***Examples****: drill, pink, bank, head* |
| **Stream 9** | **Focus:** Word families and double consonants: ock, uck, ack, ss  ***Example:*** *block, chess duck, sack* |
| **Stream 10** | **Focus:** Double consonants and consonant digraphs: ck, ff, th  ***Example:*** *quick, puff, think* |
| **Stream 11& 12** | **Focus:** Long e sound**:** ee, ea,  ***Example:*** *bee, seat, cheek, stream* |
| **Stream 13** | **Focus:** Long i sound: “y”, ie, igh, ight, i_e  ***Example:*** *fly, high, light, tied, slide* |
| **Stream 14** | **Focus:** Long u sound: ue, oo, ew  *Example: zoo, rule, few, blue* |
| **Stream 15** | **Focus:** R controlled vowels: ar, eer, ear  ***Example:*** *card, farm, clear, reindeer* |
| **Stream 16** | **Focus:** Long a sound: ay, ai, a_e  ***Example:*** *say, cane, snail, weigh, they* |
| **Stream 17** | **Focus:** Long o sound: ow, oa, oe o_e  ***Example:*** *go, yellow, boat, vote, told* |
| **Stream 18** | **Focus:** R controlled vowels and variant vowels: ore, aw, all, alk, ought  **Example:** chore, draw, wall, talk, short, thought |
| **Stream 19** | **Focus**: Diphthongs: ow, ou  ***Example:*** *now, brown, house, cloud* |
| **Stream 20** | **Focus:** Diphthongs and R controlled vowels: oy, oi, air, are, ear  ***Example:*** *boy, soil, chair, pear, where* |
| **Stream 21** | **Focus :** Suffixes and vowel digraphs: oo, -ful  ***Example:*** *book, foot, careful* |
| **Stream 22** | **Focus:** R controlled vowels: ir, ur, er, or  ***Example:*** *her, shirt, fern, worn, hurt* |
| **Stream 23** | **Focus:** Consonant digraphs: wh, kn, (sh/ch review)  ***Example:*** *which, want, knock, sheep, chop, know* |
| **Stream 24** | **Focus:** Consonant digraphs and soft ‘g’: wr, mb, j/g, dge  ***Example:*** *wrist, comb, joke, large, judge* |
| **Stream 25** | **Focus:** Consonant digraphs and double consonants: ph, zz  ***Example:*** *dolphin, photo, pizza, puzzle, sneeze* |

| **Video Demos** |
| --- |

- [**https://www.youtube.com/watch?v=5VY0sfxzXBE**](https://www.youtube.com/watch?v=5VY0sfxzXBE)
- [**https://www.youtube.com/watch?v=8gKLybOCZIQ**](https://www.youtube.com/watch?v=8gKLybOCZIQ)
- [**https://www.youtube.com/watch?v=ztz-viDKSDc**](https://www.youtube.com/watch?v=ztz-viDKSDc)
